# Supplementary material for: Generation of Vaccine Candidate Strains That Antigenically Match Classical Swine Fever Virus Field Strains
Source: Vaccines (Basel). 2025 Feb 14;13(2):188. doi: 10.3390/vaccines13020188 (PMC11860266; doi:10.3390/vaccines13020188)
Supplement: Supplementary file 1 [file vaccines-13-00188-s001.zip › Figure S1 S2 S3 S4 S5 S6_Original.pdf]

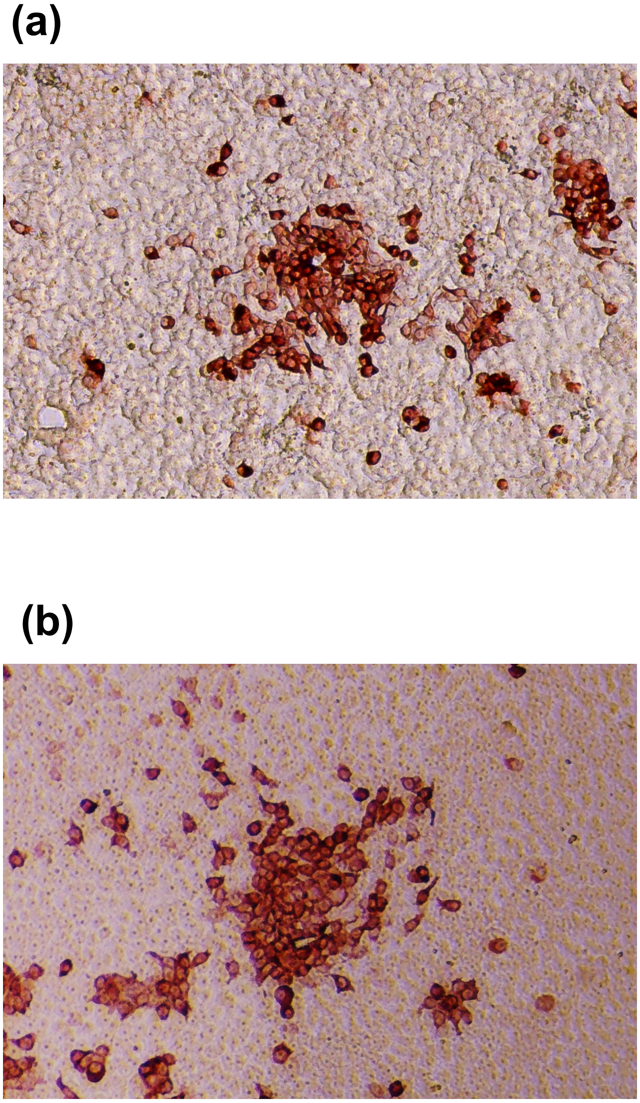

Figure S1. Rescue of (a) vGPE^{-}/HiBiT/Mie E2 and (b) vGPE^{-}/HiBiT/Mie E2/PAPeV E^{rns}. SK-L cells were electroporated with RNA transcribed from infectious cDNA clones, pGPE^{-}/HiBiT/Mie E2 and pGPE^{-}/HiBiT/Mie E2/PAPeV E^{rns}. After 3 days of incubation, cells were heat-fixed and immunostained with anti-NS3 MAb.

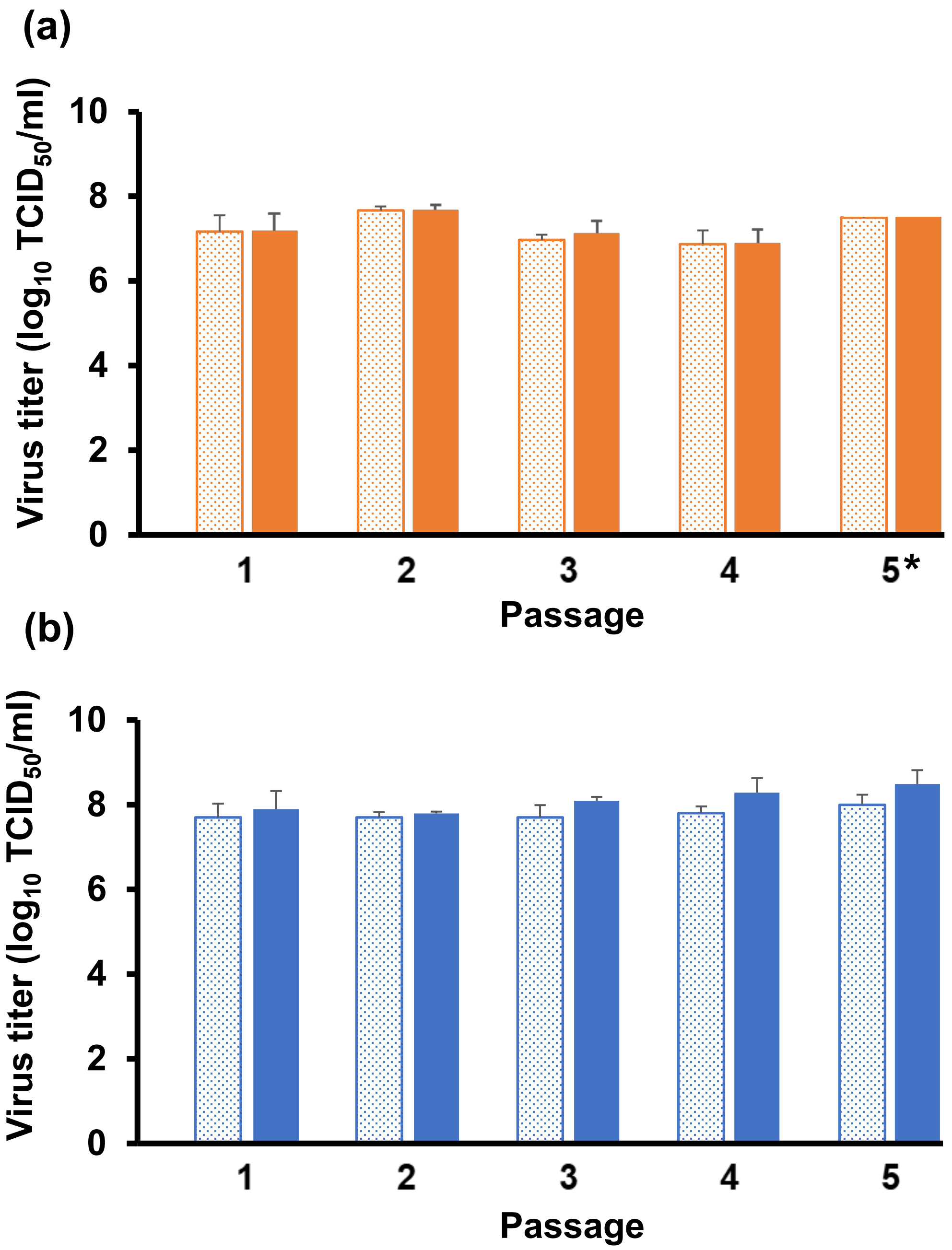

Figure S2. Growth of the recombinant viruses *in vitro*. (a) vGPE$^-$/HiBiT/Mie E2 and (b) vGPE$^-$/HiBiT/Mie E2/PAPeV E^{rns} were passaged independently in SK-L cells five times. Confluent SK-L cells were inoculated with each virus, and cell culture supernatants were collected after 3 days of incubation. The supernatants were subjected to the next passage and virus titration. Virus titration was measured by luciferase assay (dot column) and IPX (colored column). The column shows the mean value of virus titers, with error bars representing SDs (n=3). *: SDs of passage no. 5 of vGPE$^-$/HiBiT/Mie E2 (n=2) were not determined.

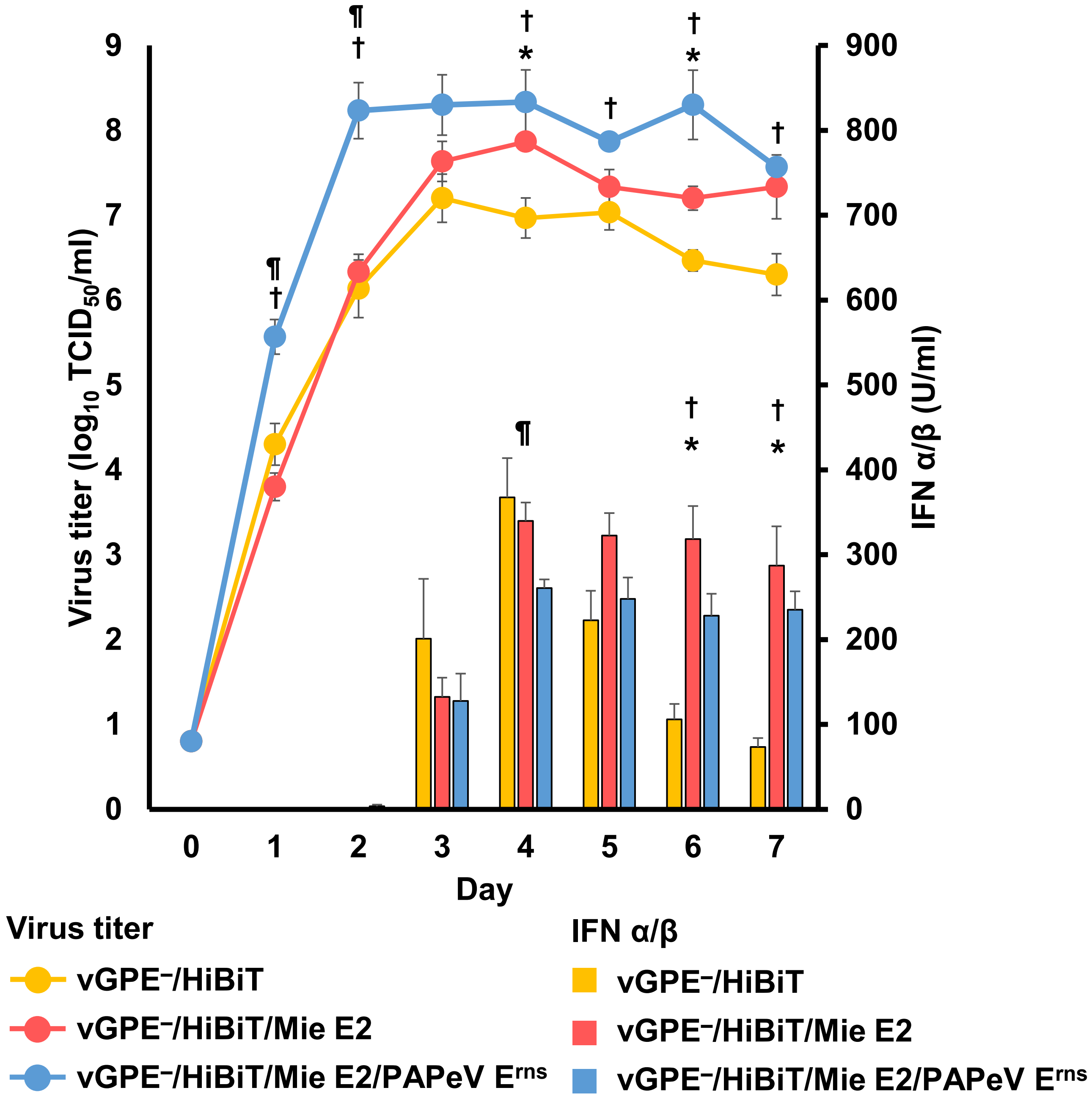

Figure S3. *In vitro* growth kinetics and IFN-α/β induction in SK-L cells inoculated with vGPE$^-$/HiBiT, vGPE$^-$/HiBiT/Mie E2, and vGPE$^-$/HiBiT/Mie E2/PAPeV E^{rns} at MOI=0.001. The significance of the differences was calculated using one-way ANOVA, followed by Student's t-test with Bonferroni correction. *$p < 0.05$ between vGPE$^-$/HiBiT and vGPE$^-$/HiBiT/Mie E2. †$p < 0.05$ between vGPE$^-$/HiBiT and vGPE$^-$/HiBiT/Mie E2/PAPeV E^{rns}. ¶$p < 0.05$ between vGPE$^-$/HiBiT/Mie E2 and vGPE$^-$/HiBiT/Mie E2/PAPeV E^{rns}.

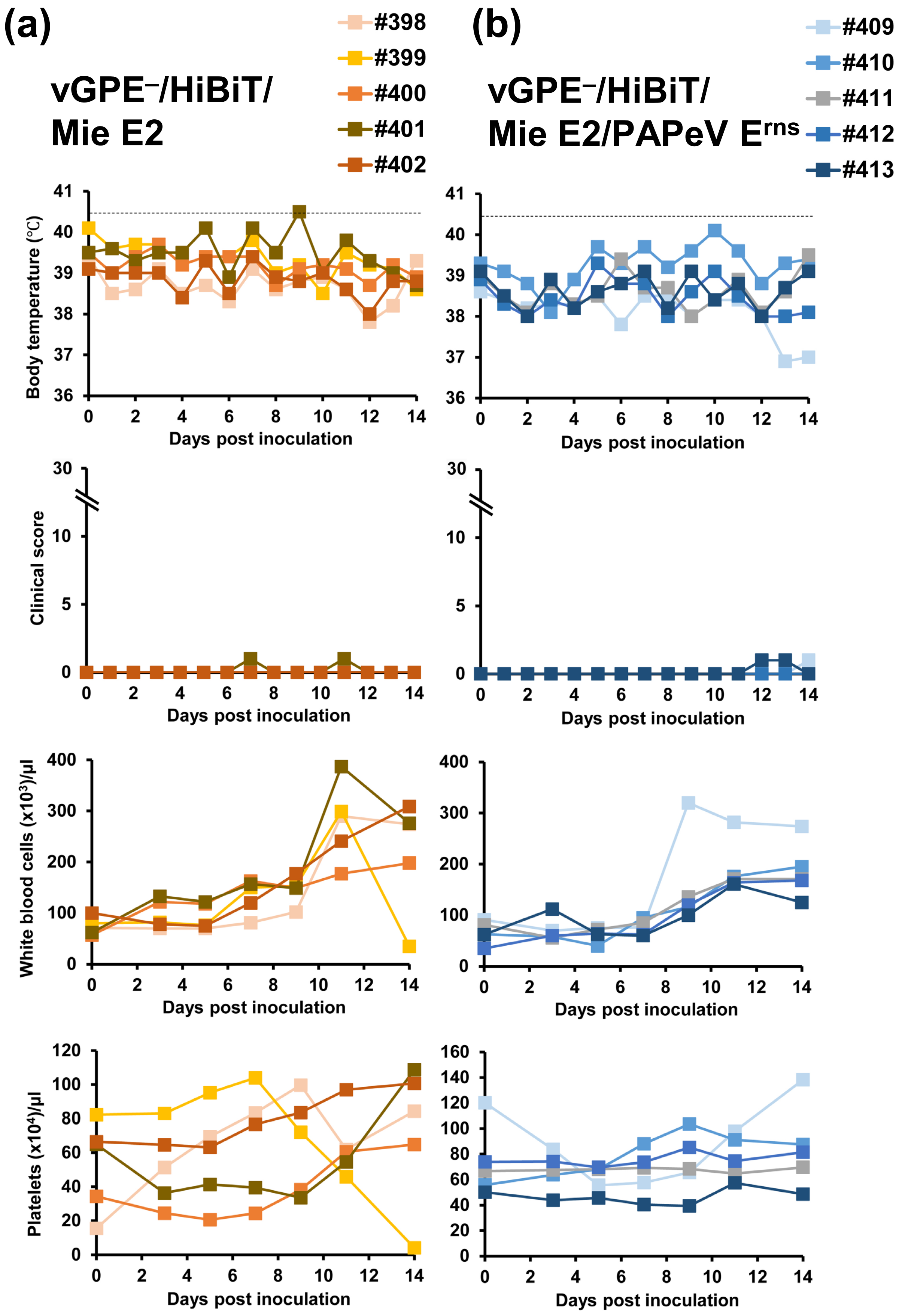

Figure S4. Body temperature, clinical score, WBC counts, and platelet counts of pigs inoculated with (**a**) vGPE⁻/HiBiT/Mie E2 and (**b**) vGPE⁻/HiBiT/Mie E2/PAPeV E^{rns}. Five 2-week-old pigs were inoculated with vGPE⁻/HiBiT/Mie E2 or vGPE⁻/HiBiT/Mie E2/PAPeV E^{rns} independently. Body temperature and clinical score were monitored daily. A dotted line indicates 40.5°C. The WBC and platelet counts were measured at each time point.

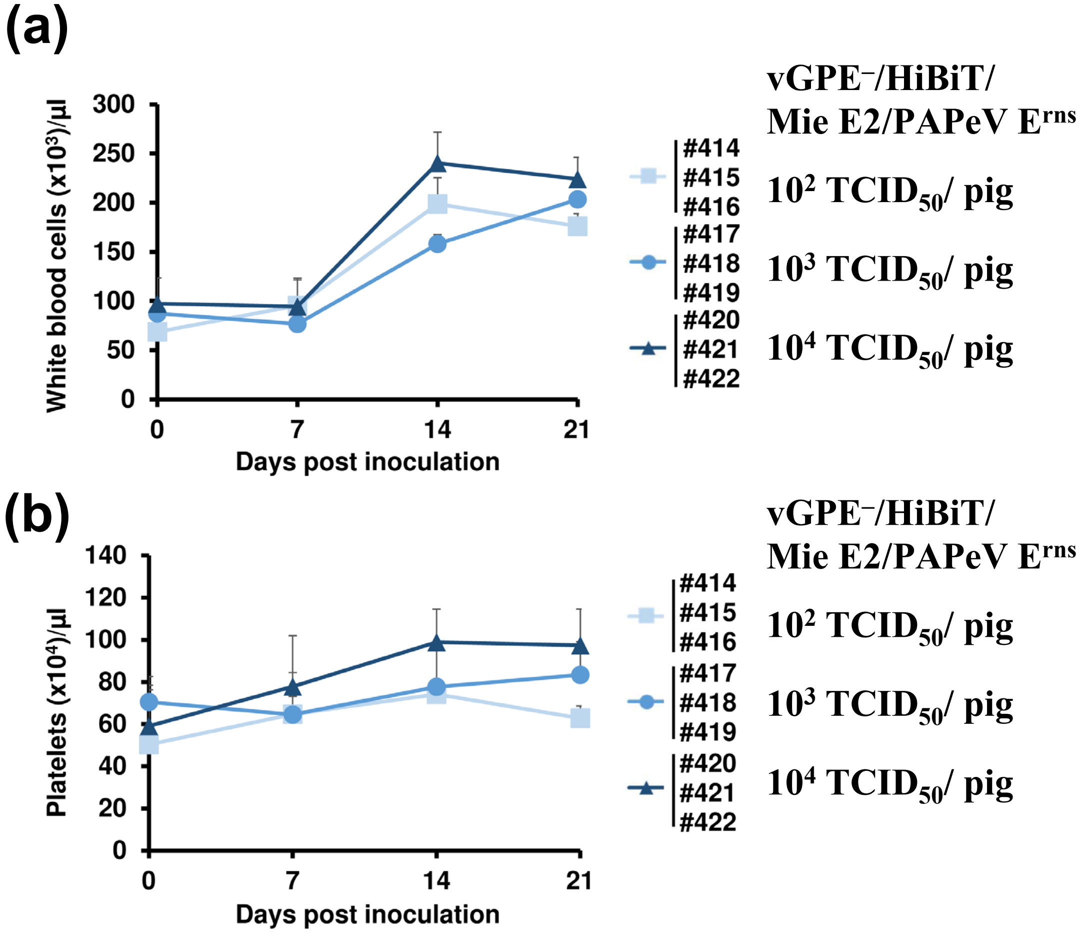

Figure S5. WBC and platelet counts in pigs inoculated with different doses of vGPE$^-$/HiBiT/Mie E2/PAPeV E^{rns}. Nine 2-week-old pigs were inoculated with vGPE$^-$/HiBiT/Mie E2/PAPeV E^{rns}, and blood samples were collected at 0, 7, 14, and 21 dpi. (**a**) WBC and (**b**) platelet counts were measured at each time point. Data are the mean, with error bars representing SDs.

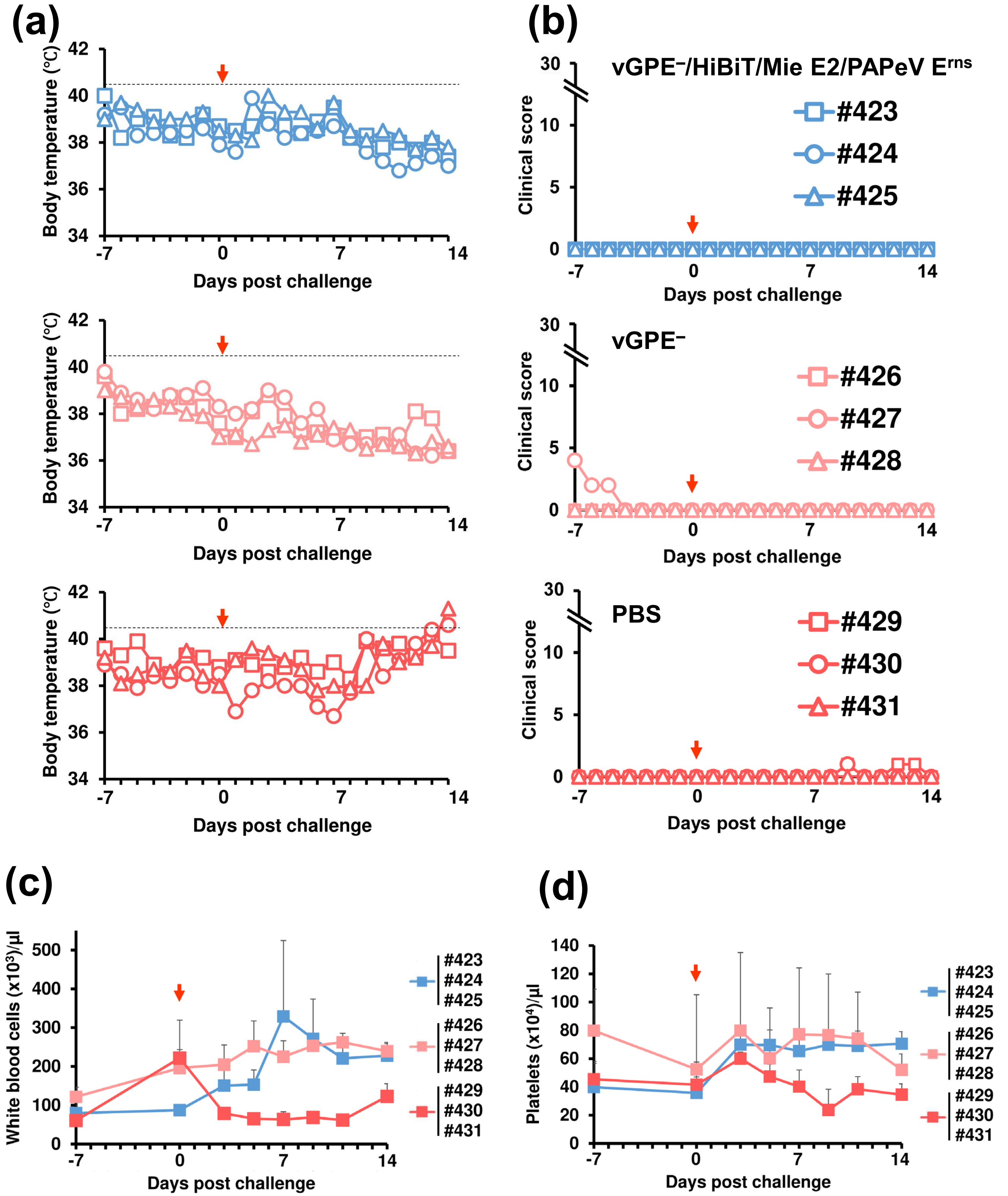

Figure S6. Body temperature, clinical score, WBC counts, and platelet counts of piglets in the challenge study. Each group of three piglets was vaccinated with vGPE⁻/HiBiT/Mie E2/PAPeV E^{rns}, vGPE⁻, and PBS, respectively, and challenged with CSFV/wb/Jpn-Mie/P96/2019. (**a**) Body temperature and (**b**) clinical score were monitored daily. An arrow and a dotted line indicate the day of the challenge and 40.5°C, respectively. (**c**) WBC and (**d**) platelet counts were measured at each time point.
