# Supplementary material for: Generation of Vaccine Candidate Strains That Antigenically Match Classical Swine Fever Virus Field Strains
Source: Vaccines (Basel). 2025 Feb 14;13(2):188. doi: 10.3390/vaccines13020188 (PMC11860266; doi:10.3390/vaccines13020188)
Supplement: Supplementary file 1 [file vaccines-13-00188-s001.zip › Table S1 S2 S3_Original.pdf]

Table S1. Primers for the plasmid construction

Plasmid	Primer	Direction*	Sequence (5′→3′)
$pGPE^{-}$/HiBiT/Mie E2	Mie-E2-F	F	ACTGGGGCACAAGGCCGGCTGTCCTGTAAGGAAGA
	Mie-E2-R	R	CTGGCCCAATGGTAAACCGGCAGCAAGTTGCTCTGTTAG
	GPE-E2rec-F	F	TTACCATTGGGCCAGGGTGAGGTAGTGTTGATAGGG
	GPE-E2rec-R	R	GCCTTGTGCCCCAGTCACTAGTAGCAGCCATATCACACC
$pGPE^{-}$/HiBiT/Mie E2/PAPeV E^{rns}	PAPeV-E^{rns}-F-HiBiT	F	GGCGGCTCGAGCGGTGTGAATATAACTCAGTGGAACCTGG
	PAPeV-E^{rns}-R	R	ACAGTAAGGTGATAGTGCCTGTGCCCCAAACCATG
	GPE-HiBiT-E^{rns}-recF	F	CTATCACCTTACTGTAATGTAACAAGC
	GPE-HiBiT-E^{rns}-recR	R	CTATCACCTTACTGTAATGTAACAAGC

* "F" indicates forward, and "R" indicates reverse.

Table S2. Primers for the differentiation between vaccine and challenge strains

Vaccine strain	Challenge strain	Target virus	Primer	Direction*	Sequence (5′→3′)	Target region
vGPE⁻ /HiBiT/Mie E2/PAPeV E^{rns}	CSFV/wb/Jpn-Mie/P96/2019	vGPE⁻ /HiBiT/Mie E2/PAPeV E^{rns}	PAPeV-E^{rns}-F-HiBiT	F	Shown in Table S1	Partial E^{rns} gene of PAPeV
			PAPeV-E^{rns}-1631R	R	TTGTGGGTTGATTGCGGGCC	
		CSFV/wb/Jpn-Mie/P96/2019	pBR322FW-4	F	CAAACATGGATGGTGTAAC	Partial E^{rns} gene of CSFV/wb/Jpn-Mie/P96/2019
			PrimerA-R5	R	TAGGTACAGAGCCGTGTCC	
vGPE⁻	CSFV/wb/Jpn-Mie/P96/2019	vGPE⁻	pBR322FW-7	F	ATGTGTGAAAGGTGAACCA	Partial E2 gene of vGPE⁻
			Mie-E2-R	R	Shown in Table S1	
		CSFV/wb/Jpn-Mie/P96/2019	MieE2-pBR322FW-7	F	CCTGTTCTACTGCAAGTGG	Partial E2 gene of CSFV/wb/Jpn-Mie/P96/2019
			Mie-E2-R	R	Shown in Table S1	

* "F" indicates forward, and "R" indicates reverse.

Table S3. Amino acid substitutions in E2 related to pathogenicity of CSFVs

Amino acid position in E2	Substitutions	Virulence: Increase↑ Decrease↓	Amino acid		Associated phenotypes	Reference
			vGPE⁻	CSFV/wb/Jpn-Mie/P96/2019		
745	T→I	↑	T	I	Increased infectious virus production and replication *in vitro*	Wu *et al*., 2016
979	M→K	↑	R	R	Enhanced pathogenicity *in vivo* Increased infectious virus production and replication *in vitro*	
830	T→A	↑	T	A	Enhanced the viral spreading *in vitro* Increased viral replication *in vivo*	Tamura *et al*., 2012
805	N→A	↓	N	N	Small-plaque formation *in vitro* Attenuation *in vivo*	Rasatti *et al*., 2006
988	A→T	↓	T	A	Decreased viral replication and E2 dimerization *in vitro* Attenuation *in vivo*	Yongfeng Li *et al*., 2022
763	S→L	↓	L	P	Delayed progress of disease, less efficient replication *in vivo* in combination with P968H	Ulrik *et al*., 2014
968	P→H		L	P		
